# Efficacy of high zinc biofortified wheat in improvement of micronutrient status, and prevention of morbidity among preschool children and women - a double masked, randomized, controlled trial

**DOI:** 10.1186/s12937-018-0391-5

**Published:** 2018-09-15

**Authors:** Sunil Sazawal, Usha Dhingra, Pratibha Dhingra, Arup Dutta, Saikat Deb, Jitendra Kumar, Prabhabati Devi, Ashish Prakash

**Affiliations:** 1 Center for Public Health Kinetics, 214A, Vinoba Puri, Lajpat Nagar-II, New Delhi, 110024 India; 2 Department of International Health, Johns Hopkins Bloomberg School of Public Health, Baltimore, MD USA; 3 Department of Pediatrics, Subharti Medical College, Meerut, Uttar Pradesh India

**Keywords:** Zinc, Biofortification, Children, Woman of child-bearing age, Morbidity, Plasma zinc, India

## Abstract

**Background:**

Biofortification of staple food crops with zinc (Zn) can be one of the cost-effective and sustainable strategies to combat zinc deficiency and prevent morbidity among the target population. Agronomic approaches such as application of Zn fertilizers to soil and/or foliar spray seem to be a practical tool for Zn biofortification of wheat. However, there is a need to evaluate its efficacy from randomized controlled trials. This study aimed to evaluate the efficacy of zinc biofortified wheat flour on zinc status and its impact on morbidity among children aged 4–6 years and non-pregnant non lactating woman of child bearing age (WCBA) in Delhi, India.

**Methods:**

In a community based, double-masked randomized controlled trial, 6005 participants (WCBA and child pairs) were enrolled and randomly allocated to receive either high zinc biofortified wheat flour (HZn, 30 ppm zinc daily) or low zinc biofortified wheat flour (LZn, 20 ppm zinc daily) for 6 months (WCBA @ 360 g/day and children @ 120 g/day). Baseline and endline blood samples were obtained for assessing hematological markers; zinc status and data on compliance and morbidity were collected.

**Results:**

Compliance rates were high; ~ 88% of the WCBAs in both the groups consumed 50% or more of recommended amount of biofortfied wheat flour during the follow up. Similarly 86.9% children in HZn and 87.5% in LZn consumed 50% or more of recommended wheat flour intake. There was no significant difference in mean zinc levels between the groups at end study. This observation might be due to a marginal difference in zinc content (10 ppm) between the HZn and LZn wheat flour, and a short intervention period. However a positive impact of bio-fortification on self-reported morbidity was observed. Compared to children in LZn group, children in HZn group had 17% (95% CI: 6 to 31%, *p* = 0.05) and 40% (95% CI: 16 to 57%; *p* = 0.0019) reduction in days with pneumonia and vomiting respectively. WCBA in the HZn group also showed a statistically significant 9% fewer days with fever compared to LZn group.

**Conclusions:**

Biofortified wheat flour had a good compliance among children and WCBAs. Significant improvement on some of the self-reported morbidity indicators suggests that evaluating longer-term effects of biofortification with higher grain zinc content would be more appropriate.

**Trial registration:**

http://ctri.nic.in/Clinicaltrials/, CTRI/2014/04/004527, Registered April 7, 2014.

**Electronic supplementary material:**

The online version of this article (10.1186/s12937-018-0391-5) contains supplementary material, which is available to authorized users.

## Background

Micronutrient malnutrition has been recognized as a huge and rapidly growing public health problem around the globe [1, 2]. More than two billion people worldwide suffer from micronutrient deficiency (especially of zinc and iron) and zinc ranks fifth in the leading risk factors for illness and disease in developing countries with high mortality [3]. Estimates indicate that approximately 20.5% of the world’s population might be at risk of zinc deficiency [4]. In India, estimated 25.9% population is at risk of inadequate zinc intake, and is considered to be high-risk category [3]. Risk factors include insufficient dietary intake due to poverty, food taboos and vegetarian diets that are high in phytate and fiber, poor utilization due to environmental factors such as poor hygiene that lead to increased infections and infestations, adverse zinc iron interaction [5] and genetic causes [6].The dietary intake of preschool children in the urban slums of India is no better than those of rural population of India [7].Women of reproductive age (WRA) are also vulnerable to nutritional deficiencies, including that of zinc [8]. Poor maternal zinc status has been associated with negative pregnancy outcomes [9], including spontaneous abortion, congenital malformation, low birth weight, and preterm delivery [10, 11]*.*

Micronutrient deficiencies can be alleviated by direct (nutrition-specific) and indirect (nutrition-sensitive) interventions [12]. Direct interventions focus on consumption behavior and include dietary diversification, micronutrient supplementation, modification of food choices and fortification. Nutrition-sensitive interventions address the underlying determinants of malnutrition and include biofortification. Biofortification is the process of increasing the content and/or bioavailability of essential nutrients in crops during plant growth through genetic and agronomic pathways [13]. Genetic biofortification involves either genetic engineering or classical breeding [14]. Agronomic biofortification is achieved through micronutrient fertilizer application to the soil and/or foliar application directly to the leaves of the crop. Even though this approach seems simple and inexpensive, this strategy has been successful in only limited cases and in particular geographical locations due to the limitations of fertilizer and soil chemistry, together with the added complications of nutrient mobility and storage within the plant.

Although zinc supplementation has been proven to be cost effective strategy in reducing morbidity and improving growth, giving zinc supplementation daily is not feasible as a program [15] thus, other sustainable methods such as food based intervention to increase zinc intakes need to be explored and evaluated. Plant breeding of staple crops, such as wheat or rice, to increase the zinc concentration can be a promising approach to increase zinc intakes of populations in developing countries [16, 17]. In northern India, although the consumption of cereals has declined in the past few decades, wheat consumption has shown no significant change and has the highest dietary share; contributing more than 50% calories to the diet [18], which is similar to consumption in many developing countries [19]. Thus, biofortification of wheat can be one of the novel food based approaches, which can provide higher levels of micronutrients directly into key staple foods [19–21].

Recently, Harvest Plus and its partners have developed wheat lines that can achieve zinc concentration of 60–70 ppm when adequately fertilized with zinc, adding ~ 20–25 ppm of zinc in the daily diet of children (aged 4–6 years) and women of reproductive age. However, there is lack of scientific evidence from randomized controlled trials for its efficacy in improving zinc nutritional status and related health outcomes in the target population groups. Therefore, we evaluated the efficacy of high zinc biofortified wheat flour on zinc status, prevention of morbidity and growth among children aged 4–6 years and non pregnant non lactating women aged 15–49 years compared to group that received low zinc biofortified wheat flour.

## Methods

### Study population and setting

This study was carried out in Sangam Vihar, a resettlement colony in outskirts of south Delhi; and Harsh Vihar, a semi-urban locality in North East District, Delhi, India. Entire study area at both field sites has been digitized and all the households are geo referenced and numbered. Both sites have similar demographic profile. Most of the inhabitants are migrants from eastern Uttar Pradesh, Bihar and Rajasthan. Literacy rates are low with 50% of the women being illiterate. About 80% of the men work as daily wage laborers or in factories, while 95% of women are housewives. Average family income is below US$600/year. Community has minimal access to sewage, drinking water and paved roads. Diarrhea and respiratory illnesses are common causes of childhood mortality and morbidity. Field sites are representative of wheat eating population of India.

### Ethics

The human research and ethical review committees at the Johns Hopkins University, USA; Annamalai University, Tamil Nadu, India and Subharti Medical College, Meerut, UP, India approved the study protocol. The purpose of the study was explained to the participants/parents (in case of children) in the local language, and a written informed consent was obtained.

### Sample size

Sample size calculations were based on prevalence of zinc deficiency of 70% (assessed by plasma zinc levels) in previously conducted studies in the same population [22]. A sample size of 3000 (1500 in each of the intervention and placebo groups) each for WCBA and children was sufficient to detect a clinically important difference of 25% between a group 1 proportion, of 0.400 and a group 2 proportion, of 0.455 (odds ratio of 1.250), using a two-sided χ^2^test with 80% power, at 5% significance level and assuming 10% attrition rate.

### Consent and enrollment

#### Eligibility criteria

##### Preschool children

Between 4 and 6 years of age, not severely malnourished requiring rehabilitation; parental permission to participate; likely to live in the study area for at least 6 months, wheat as a staple diet.

##### Women of child-bearing age

Between 15 and 49 years of age (non-lactating and non pregnant), not having any severe illnesses requiring hospitalization; consent to participate; permanent residents or staying in the study area for next six months, wheat as a staple diet.

The study area was divided into small working areas (grids) and all the households in the grid were allocated to a study worker. The study worker visited every household in the grid allocated to him/her to identify the mother-child pairs eligible to participate in the study. After identification of potential participants, the study supervisor visited the household and explained the study purpose to the participants/caregivers (in case of child). All potential participants were requested to come to the clinic for formal consent and enrollment into the study. In the clinic, study physician examined the participant (WCBA and child) for eligibility criteria and if successful, the study supervisor obtained the formal consent from the participant/spouse (in case of woman) or parents (in case of children) and enrolled the participants into the study.

### Masking and randomization

Households with eligible mother and child pairs were randomly allocated to receive either high zinc biofortified wheat or low zinc biofortified wheat. For masking purposes, wheat bags were labeled with alphanumeric codes. Eight codes (4 for each intervention group) were used. Each of these 8 codes had a letter i.e. A through H in the beginning. These codes were only known to the manufacturer or person doing the packaging of the interventions and to the data safety and monitoring board (DSMB) members. These codes were maintained in a sealed envelope till the study was over. A computerized randomization schedule with permuted block length of 16 was generated for 6050 participants.

### Intervention

Harvest Plus provided high zinc biofortified and low zinc biofortified wheat flour free of charge for the entire duration of the project. The wheat variety used for control and intervention groups was a commercial variety (PBW 550) developed and released by Punjab Agricultural University in 2007. Standard farming techniques as prescribed in the package and practices recommended by Punjab Agricultural University, Ludhiana were followed during the entire growing period. Low zinc biofortified wheat was grown in agro-ecological conditions that limit the uptake of soil zinc by plants and received no additional zinc fertilization. The wheat (HZn) for the intervention group was grown in agro-ecological conditions and additional foliar spraying of 0.5% zinc sulphate fertilizer that foster the zinc uptake by the plant and its deposit in the growing seeds was done. PBW 550 is not a genetically modified organism (GMO) and was grown at farmers’ fields in Punjab (districts Kapurthala and Bathinda), India. The wheat was milled and packaged by Arti Roller Flour Industries Ltd.- an ISO 22000:2005 certified company.

### Intervention delivery and follow up

Participants were provided wheat flour ration every 25 days based on the dietary intake data available from the previously conducted studies and the qualitative data collected on wheat consumption patterns during the preparatory phase of the study. They were clearly explained and demonstrated that they could use the biofortified wheat flour for making chapattis, puris, porridge etc. as with regular wheat flour. The cooked preparation (e.g. *chapatti/tortilia or puri*) could be consumed as such or could be eaten with any lentil or vegetable. Participants were encouraged to take normal home diet ad libitum. During the follow-up, a designated ration (based on the findings from the preparatory phase) was supplied to the enrolled participants by the study staff with an advice to consume 120 g (aged 4–6 years) or 360 g (aged 15–49 years) of assigned wheat flour daily. To prevent sharing of ration with other family members, additional 200 g wheat flour daily per household was provided to the family in both the groups. Wheat flour for family members was same as was given to the participant (HZn or LZn). Participants were followed up on a weekly basis by the study worker for collection of compliance, morbidity information and medication/supplement use. On each visit, the compliance (adherence with the intervention) was assessed by collecting the information on reported intake of biofortified wheat flour in terms of number of days it was being offered and consumed, portion size consumed and unused quantity recovered. The participant/parent of the child was given a pictorial diary card and was asked to record the quantity of wheat flour preparation consumed on a daily basis. Study worker reviewed the diary card on each visit, collected the filled diary card and left a new card for recording information.

If a participant was found to be sick during follow-up, he or she was referred to the study clinic for a detailed morbidity assessment by the study physician. They had been counselled to visit the clinic anytime to seek care for illnesses. The participant was provided treatment by the study physician. During the study, spot checks were carried out to assess the food availability at home. During the follow up, on a sub-sample of participants, dietary data were collected using an interactive 24-h dietary recall method [23] to estimate the macro- or micro-nutrient intake with special emphasis on zinc, iron and vitamin A and antinutrient (phytic acid) content by the trained nutritionist at home.

### Data collection

#### Socio-demographic data collection

Socioeconomic status, demographic details, illness information in the last two weeks was recorded in a pre-designed data-recording sheet at the clinic for baseline data collection.

#### Baseline assessment

After obtaining the informal consent, participants were asked to visit the clinic on a scheduled date for baseline assessments.

##### Medical examination

The study physician conducted a physical examination and collected information on the medical history of the participant for the last two weeks including history of hospitalizations/blood transfusions in the last 6 months.

##### Anthropometry measurements

Two independent observers measured weight to the nearest 10 g with an electronic scale (ATCO weighing solutions, India) and height to the nearest 0.1 cm using locally improvised height board. In addition, waist of the WRA was measured with a tape measure one inch above the navel while standing and values reported to the nearest quarter inch.

##### Blood sampling, plasma separation and laboratory procedures

A venous blood sample (5 ml) was collected in trace element-free syringe by trained technician. Approximately half ml of blood was transferred to an EDTA tube for hemogram and the remaining blood was transferred to a tube containing zinc free heparin, for separation of plasma*.* Approx 100 μL of EDTA whole blood was used to determine haemoglobin, RDW, MCV, MCH by a KX21 automated haematological analyzer (Sysmex tech, Japan).

##### Plasma zinc

Plasma was separated within 15 min of blood collection and aliquots transferred into trace element-free Eppendorf plastic tubes for storage at − 20 °C until analysis.

#### Endline assessment

Blood sample and anthropometry assessments were repeated at the end of 6 months of follow up.

#### Laboratory analysis

Plasma samples were analyzed for zinc status using an atomic absorption spectrometer (AAS 400-Perkin Elmer, USA) at the micronutrient research lab in Subharti Medical College, Meerut. For quality assurance, we ran three in house pooled plasma controls (low, medium and high) after every 10 batch of samples.

### Zinc and phytate analysis of wheat flour samples

Biochemical analysis was carried out on all 18 batches of wheat flour received for zinc and phytate content (Additional file 1: Table S1 and Table S2). From batch-9 onwards, zinc analysis was undertaken in one additional lab, in order to ensure the reliability of the test results of wheat flour samples. The zinc content of high zinc biofortified wheat flour was 30 ppm and of low zinc biofortified wheat flour was 20 ppm.

### Statistical analyses

We used in house data management system with stringent range and logical checks for data entry and management. Study team used netbooks to enter real time data. The analyses were performed in STATA 13.0 (Stata Corp., College Station, Texas, USA) and SPSS 23 (SPSS Inc., Chicago, Illinois, USA). Intent-to-treat analysis was performed and all participants were included in analysis irrespective of compliance to intervention. For the primary analysis the authors used alphabetical codes for the groups as they were still blinded to the real group identity. The groups were assessed for comparability at baseline. The statistical significance for the group differences of continuous variables was established using a Student’s t-test, and of categorical variables by chi-square test. Significance was considered as *p*-values < 0.05. Participants were considered zinc deficient if their plasma zinc levels were below 70 μg/dL. They were further classified into 3 categories; improved: if they were zinc deficient at baseline but became zinc sufficient at endline; worsened: if sufficient at baseline but became deficient at endline, and no change: if there was no change in status. Risk estimates and confidence intervals were calculated for morbidity indicators for each participant; days with morbidity were considered as numerator while days’ informant available was considered as denominator.

## Results

### Participants

**Fig. 1:**
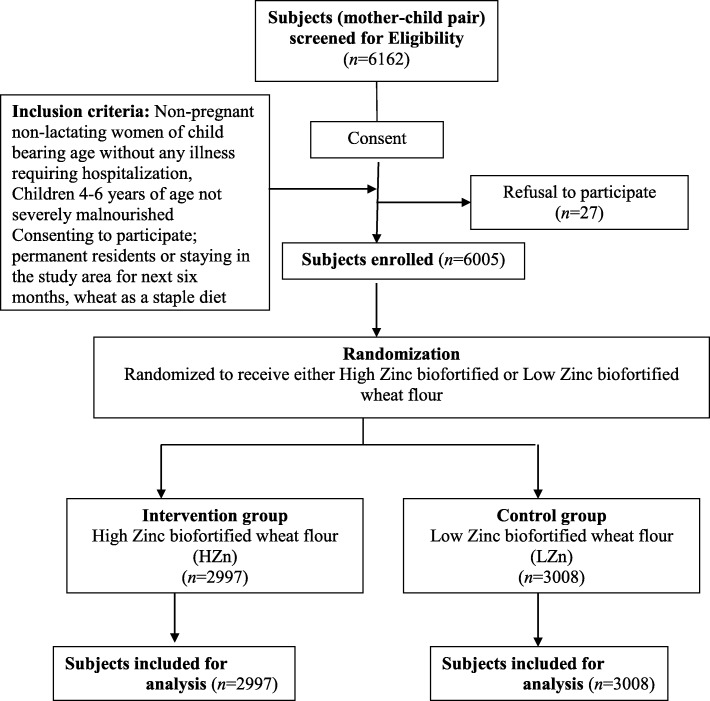
Study participants flow diagram

Between April 14, 2014 and February 23, 2015, a total of 6162 participants were screened, 157 failed eligibility criteria and a total of 6005 participants were enrolled. The enrolled participants were randomly allocated to receive either high zinc biofortified (HZn) wheat flour (*n* = 2997) or the low zinc biofortified (LZn) wheat flour (*n* = 3008) (Fig. 1).

### Baseline data

Both groups had comparable socio-economic, and demographic profile (Table 1). Mean age at enrollment for WCBA was 29.8 (SD: 5.9) years in HZn and 29.8 (SD: 6.0) in LZn group. Children at enrollment had mean age of 5.0 (SD: 0.8) years in HZn and 5.1 (SD: 0.8) years in LZn group.

**Table 1:** Baseline characteristics of the study population

	HZn	LZn
Age (years)		
WCBA	29.8 ± 5.9	29.8 ± 6.0
Children	5.0 ± 0.8	5.1 ± 0.8
Type of family		
Nuclear	83.1%	80.9%
Type of household		
Pucca	27.8%	26.2%
Kuccha-Pucca	71.3%	73.3%
Kuccha	0.9%	0.5%
Own house	52.9%	56.3%
Tube well/bore water	86.0%	84.7%

### Compliance

Compliance was high in both groups. Women in both groups consumed >= 50% of recommended amount of biofortified wheat flour (360 g) on 88% of the follow up days (HZn: 88.1%; LZn: 88.7%). Similarly 86.9% children in HZn and 87.5% in LZn consumed >= 50% of recommended amount of biofortified wheat flour (120 g) during follow up (Table 2).

**Table 2:** Compliance of biofortified wheat flour among WCBA and children

WCBA	HZn (*n* = 287,514)	LZn (*n* = 286,605)
Days consumed >= 50% of recommended wheat flour	253,254 (88.1)	254,100 (88.7)
Days consumed full (360 g daily)	159,569 (55.5)	161,579 (56.4)
Days consumed more than half	79,432 (27.6)	76,200 (26.6)
Days consumed half	14,253 (5.0)	16,321 (5.7)
Days consumed 1/4th	1087 (0.4)	1051 (0.4)
Days did not consume	33,173 (11.5)	31,454 (11.0)
Children	HZn (*n* = 288,903)	LZn (n = 286,901)
Days consumed >= 50% of recommended wheat flour	251,214 (86.9)	250,981 (87.5)
Days consumed full (120 g daily)	157,547 (54.5)	155,076 (54.1)
Days consumed more than half	71,891 (24.9)	73,714 (25.7)
Days consumed half	21,776 (7.5)	22,191 (7.7)
Days consumed 1/4th	2257(0.8)	2274 (0.8)
Days did not consume	35,432 (12.3)	33,646 (11.7)

### Effect on hematological markers

At baseline 18.0% children in HZn and 16.8% in LZn group were moderately anemic (70–100 g/L); while among WCBA’s 19.6% in HZn and 21% in LZn were moderately anemic. At the end of 6 months of follow up, children in HZn and LZn group had mean hemoglobin levels (g/L) of 114.0 ± 15.7 and 114.6 ± 16.1 respectively; 13.5% in HZn and 11.9% in LZn remained moderately anemic at endline sampling. At endline, HZn WCBA’s had mean hemoglobin levels (g/L) of 114.1 ± 19.8 while LZn WCBA’s had 113.2 ± 19.9. There was a statistically significant difference in RDW between HZn and LZn children at endline but this seems to be by chance since RDW without anemia has no predictive value for iron deficiency anemia [24]. Comparison of hematological markers between the two groups at baseline and after 6 months of intervention is presented in Tables 3 and 4.

**Table 3:** Baseline and endline hematological parameters among WCBA (HZn vs. LZn)

	HZn	LZn	Difference of means (95% CI)	*p* value
Baseline	(*n* = 1400)	(*n* = 1388)		
Hb (g/L)	113.1 ± 20.6	112.8 ± 20.8	0.6 (−0.9 to 2.1)	0.5
Hct (%)	35.5 ± 5.8	35.4 ± 5.8	0.1 (− 0.3 to 0.5)	0.6
MCH (pg)	27.9 ± 3.8	27.7 ± 4.0	0.2 (−0.07 to 0.5)	0.1
MCV (fl)	87.6 ± 9.7	87.1 ± 10.2	0.5 (−0.2 to 1.3)	0.2
RDW (%)	15.8 ± 2.1	16.0 ± 2.3	−0.1 (− 0.3 to 0.03)	0.1
Endline	(*n* = 1316)	(*n* = 1324)		
Hb (g/L)	114.1 ± 19.8	113.3 ± 19.8	0.8 (−0.7to 2.3)	0.3
Hct (%)	34.8 ± 5.5	34.7 ± 5.5	0.1 (−0.2to 0.5)	0.4
MCH (pg)	28.0 ± 3.8	27.9 ± 3.9	0.1 (−0.2 to 0.4)	0.4
MCV (fl)	85.7 ± 9.7	85.5 ± 9.9	0.2 (−0.6 to 0.9)	0.6
RDW (%)	15.5 ± 2.3	15.6 ± 2.2	−0.1 (− 0.2 to 0.1)	0.6

*Hb* Hemoglobin, *HCT* Hematocrit, *MCH* Mean corpuscular hemoglobin, *MCV* Mean corpuscular volume, *RDW* Red blood cell distribution width

**Table 4:** Baseline and endline hematological parameters among children (HZn vs. LZn**)**

	HZn	LZn	Difference of means (95% CI)	*p* value
Baseline	(*n* = 1383)	(*n* = 1364)		
Hb (g/L)	112.1 ± 17.1	111.7 ± 16.4	0.4 (−0.9 to 1.6)	0.6
Hct (%)	34.2 ± 4.8	34.2 ± 4.6	0.1 (−0.2 to 0.5)	0.5
MCH (pg)	25.2 ± 3.0	25.2 ± 2.8	0.0 (−0.2 to 0.2)	0.9
MCV (fl)	77.0 ± 7.6	77.0 ± 7.2	0.0 (−0.6 to 0.6)	0.9
RDW (%)	15.4 ± 2.1	15.3 ± 1.9	0.1 (−0.02 to 0.3)	0.1
Endline	(*n* = 1269)	(*n* = 1265)		
Hb (g/L)	114.0 ± 15.7	114.6 ± 16.1	−0.6 (−1.9 to 0.6)	0.3
Hct (%)	33.9 ± 4.3	34.1 ± 4.4	−0.2 (− 0.5 to 0.2)	0.3
MCH (pg)	25.6 ± 2.8	25.5 ± 2.6	0.1 (−0.2 to 0.3)	0.6
MCV (fl)	76.2 ± 7.4	76.1 ± 6.7	0.1 (−0.4 to 0.7)	0.6
RDW (%)	15.1 ± 2.3	14.9 ± 2.0	0.2 (0.05 to 0.4)	0.0

*Hb* Hemoglobin, *HCT* Hematocrit, *MCH* Mean corpuscular hemoglobin, *MCV* Mean corpuscular volume, *RDW* Red blood cell distribution width

### Effect on plasma zinc status

At baseline 85.8% of children in HZn and 84.7% in LZn had zinc levels below 70 μg/dL. After 6 months of follow up, 27.2% children in HZn and 26.7% children in LZn showed improvement (Zinc levels above 70 μg/dL) (OR: 0.98; 95% CI 0.81–1.17).There was change in plasma zinc levels from baseline to endline in both the groups (Diff in means (μg/dL):1.08; 95% CI -0.54-2.69). Among WCBAs, 85.5% in HZn group and 87.5% in LZn group were zinc deficient at baseline. Of these 21% WCBAs in HZn and 22.4% in LZn group became zinc sufficient after 6 months of intervention (OR:1.08; 95% CI 0.90–1.31) (Tables 5 and 6).

**Table 5:** Comparison of plasma zinc levels (μg/dL) among children at baseline and endline

	HZn	LZn	
*Baseline*	(*n* = 1448)	(*n* = 1445)	
Mean ± SD	55.9 ± 14.5	56.9 ± 13.9	
Median	55.5	57.4	
Zinc deficient	1242 (85.8)	1224 (84.7)	
*Endline*	(*n* = 1239)	(*n* = 1234)	
Mean ± SD	63.0 ± 15.6	63.1 ± 15.7	
Median	61.6	63.4	
Zinc deficient	862 (69.6)	855 (69.3)	
*Change in Zinc Status*			
Change in mean Zinc levels	7.22 ± 20.3	6.14 ± 20.4	1.08 (−0.54 to 2.69)
Improvement in zinc status	322 (27.2)	312 (26.7)	0.98 (0.81 to 1.17)

**Table 6:** Comparison of plasma zinc levels (μg/dL) among WCBAs at baseline and endline

	HZn	LZn	
*Baseline*	(*n* = 1472)	(*n* = 1476)	
Mean ± SD	55.4 ± 14.0	54.9 ± 14.4	
Median	55.1	55.4	
Zinc Deficient	1258 (85.5)	1292 (87.5)	
*Endline*	(*n* = 1312)	(*n* = 1317)	
Mean ± SD	60.9 ± 14.7	60.7 ± 14.8	
Median	61.6	59.9	
Zinc Deficient	994 (75.8)	989 (75.1)	
*Change in zinc status*			
Change in Mean Zinc levels	5.49 ± 19.8	5.91 ± 19.5	−0.42 (−1.92 to 1.08)
Improvement in zinc status	265 (21.0)	285 (22.4)	1.08 (0.90 to 1.31)

### Effect on morbidity

Based on self-reported morbidity information, HZn children experienced 17% lower days with pneumonia compared to children in LZn group. There was a statistically significant 39% lower risk of days with vomiting in HZn group compared to LZn group children. Days with ear discharge were 17% lower in HZn group compared to LZn group. WCBAs in HZn group had statistically significant 9% reduction in days with fever compared to WCBAs in LZn group (Tables 7 and 8).

**Table 7:** Effect of consumption of wheat flour (HZn vs. LZn) on morbidity among children

	HZn	LZn	RR	95% CI
Days with diarrhea	126	119	1.05	0.82 to 1.35
Days with pneumonia	203	244	0.82	0.68 to 0.99
Days with fever	934	960	0.96	0.88 to 1.06
Days with vomiting	60	99	0.60	0.44 to 0.83
Days with ear discharge	72	87	0.82	0.60 to 1.12

**Table 8:** Effect of consumption of wheat flour (HZn vs. LZn) on morbidity and physical activity among WCBA

	HZn	LZn	RR	95% CI
Days with fever	976	1089	0.90	0.82 to 0.98
Days with usual physical activity	250,313	250,914	1.00	0.99 to1.01
Days with usual activity performance	252,133	252,091	1.00	0.99 to1.01

## Discussion

This study, reports the first large randomized controlled trial, evaluating efficacy of zinc biofortified wheat consumption in improvement of micronutrient status, and prevention of morbidity among preschool children (aged 4–6 years) and non pregnant non lactating women of child bearing age. The study was conducted in a low resource setting in India with high levels of zinc deficiency among children and women of child bearing age.

In our study, consumption of high zinc biofortified wheat flour for 6 months compared to low zinc biofortified wheat resulted in statistically significant reduction in days with pneumonia and vomiting among children and days with fever among women of reproductive age. Similar findings have been reported by earlier zinc supplementation randomized controlled trials showing an effect of zinc supplementation in decreasing morbidity and mortality in children due to gastrointestinal and respiratory infections [25]. Participants in all the zinc supplementation studies were supplemented with zinc daily, over and above regular dietary intake and dose of zinc supplementation ranged from 10 mg to 20 mg and duration from 4.6 to 18 months.

There is limited evidence available on the efficacy of zinc biofortified crops. Rosado et al. [26] carried out a trial among 27 women who consumed 300 g of 95% or 80% extracted wheat flour of tortillas for 2 consecutive days using either biofortified (41 mg Zn/g) or control (24 mg Zn/g) wheat. Zn intake from the biofortified wheat was higher at 80% extraction compared with the corresponding control wheat (*p* = 0.007). In India, Kodkany et al. [27] provided zinc and iron biofortified pearl millet flour to children under 2 years and concluded that quantities of zinc absorbed from biofortified pearl millet as the major food staple is more than adequate to meet the physiological requirements of these nutrients. Study from Zambia [28] indicated that feeding biofortified maize could meet zinc requirements and provide an effective dietary alternative to regular maize. In contrast the grain zinc concentrations levels achieved using the agronomic Zn foliar sprays/fertilizers in our study were very low. In our study the high zinc biofortified wheat would have at best delivered 3.6 mg/d of zinc to children and 10.8 mg/d to women if they had consumed 100% of recommended intake; in comparison the control group (low zinc biofortified wheat) would have delivered 2.4 mg/d to children and 7.8 mg/d to women. The daily Zn requirement is 15 mg for both adults and children that are 4 and older [29]. To meet this allowance wheat grains should contain 40–60 mg kg^−1^Zn. Although there was an improvement in zinc status (plasma zinc levels> 70 μg/dL) within each group but given the adjuvant used to enhance zinc absorption through the leaves was not optimal and the minimum expected increase of 20 ppm over the low biofortified wheat was not achieved, this improvement was similar between the groups. This study does indicate the potential for use of biofortification as a means of improving zinc nutrition with a caveat to improve the biofortification process such that wheat grains contain 40–60 mg kg^−1^Zn to meet the recommended dietary allowance of Zn.

Zinc bioavailability pilot trial designed to estimate the amount of zinc absorbed from zinc rice compared to conventional rice using the triple stable isotope tracer ratio technique, failed to produce detectable differences in absorbed zinc [30]. High phytate and lower-than-expected zinc in the biofortified zinc variety was suggested as a cause for no significant difference in absorption between groups. Zinc uptake by wheat and translocation into the grains is affected by genetic-environment interactions and evidently by the efficiency of the foliar application of the fertilizer. In our study, the adjuvant used to enhance zinc absorption through the leaves was not optimal and could not produce the HZn variety with expected Zn content.

There is sufficient evidence from previous studies undertaken in 7 countries over different seasons [31] that foliar zinc fertilization can increase both yields and nutritional quality of crops. In these studies there was an average increase of 77% over controls (from 29 ppm to 48 ppm). This efficacy trial affirms importance of zinc biofortification with adequate levels of Zn concentration. The data suggests that there was no bioavailability issue as the plasma zinc levels in both groups did improve substantially between baseline and endline. There has to be a significant differential between the two groups in terms of grain Zn content to be able to show the difference between the groups. The study results indicate that both children and WCBAs consumed a full ration of 120 g/360 g daily only on 55% to 60% of the days, although consumption of 50% or more of recommended intake was close to 90% which further outlines the importance of achieving higher grain Zn concentrations and continuous consumption for longer duration.

### Limitation of the study

One main limitation of the study was that the differential zinc content between HZn and LZn was marginal. The study was designed with 40 ppm difference between HZn and LZn but the wheat flour provided for the study had only 10 ppm differential between the two groups. The results of the study need to be inferred in this context. The shorter duration of the study (6 months) could be another limitation for finding meaningful differences between the groups.

## Conclusions

Biofortified wheat flour had a good compliance among children and women suggesting agronomic approaches such as application of Zn-containing fertilizers could be a good delivery mechanism. Significant improvement on some of the morbidity indicators suggests that evaluating longer term effects of biofortification with higher zinc content would be more appropriate. Research programs are needed to develop and improve Zn application methods in terms of form, dose, and application time of Zn fertilizers.The drive for higher yields is usually accompanied by a dilution effect of minerals due to the additional grain starch accumulation. The scientists and breeders need to work together to achieve required grain Zn contents.

## Additional file

Additional file 1: **Table S1.** Zinc content of the study wheat flour. **Table S2.** Phytic acid content of the study wheat flour. (PDF 76 kb)

## Abbreviations

AAS: Atomic absorption spectroscopy
CI: Confidence interval
DSMB: Data and safety monitoring board
EAR: Estimated average requirements
EDTA: Ethylenediaminetetraacetic acid
GMO: Genetically modified organism
HZn: High zinc biofortified wheat flour
ISO: International organization for standardization
LZn: Low zinc biofortified wheat flour
MCH: Mean corpuscular hemoglobin
MCV: Mean corpuscular volume
RDW: Red cell distribution width
RR: Relative risk
SD: Standard deviation
SPSS: Statistical package for the social sciences
UP: Uttar Pradesh
WCBA: Woman of child bearing age
WRA: Women of reproductive age
Zn: Zinc
χ^2^: Chi Square

## References

[1] Sharma A, Patni B, Shankhdhar D, Shankhdhar SC (2013). Zinc – an indispensable micronutrient. Physiol Mol Biol Plants.

[2] Jorge EM, Wolfgang HP, Peter B (2008). Biofortified crops to alleviate micronutrient malnutrition. Curr Opin Plant Biol.

[3] International Zinc Nutrition Consultative Group (IZiNCG) (2004). Technical document #1. Assessment of the risk of zinc deficiency in populations and options for its control. The United Nations University. Food Nutr Bull.

[4] Wuehler SE, Peerson JM, Brown KH (2005). Use of national food balance data to estimate the adequacy of zinc in national food supplies: methodology and regional estimates. Public Health Nutr.

[5] Sandström B, Bügel S, McGaw BA, Price J, Reid MD (2000). A high oat-bran intake does not impair zinc absorption in humans when added to a low-fiber animal protein-based diet. J Nutr.

[6] Seshadri S. Nutritional anaemia in South Asia. In: Gillespie SK, editor. *Malnutrition in South Asia: a regional profile*. UNICEF Regional Office for South Asia. 1997. p. 75–124.

[7] Ghosh S, Shah D (2004). Nutritional problems in urban slum children. Indian Pediatr.

[8] Chandyo RK, Strand TA, Mathisen M, Ulak M, Adhikari RK, Bolann BJ (2009). Zinc deficiency is common among healthy women of reproductive age in Bhaktapur, Nepal. Nutr.

[9] Osendarp SJ, West CE, Black RE (2003). The need for maternal zinc supplementation in developing countries: an unresolved issue. J Nutr.

[10] Prasad AS (1996). Zinc deficiency in women, infants and children. J Am Coll Nutr.

[11] Seshadri S (2001). Prevalence of micronutrient deficiency particularly of iron, zinc and folic acid in pregnant women in South East Asia. Br J Nutr.

[12] Ruel MT, Alderman H (2013). Nutrition-sensitive interventions and programmes: how can they help to accelerate progress in improving maternal and child nutrition? (and the maternal and child nutrition study group). Lancet.

[13] Bouis HE, Hotz C, McClafferty B, Meenakshi JV, Pfeiffer WH (2011). Biofortification: a new tool to reduce micronutrient malnutrition. FoodNutr Bull.

[14] Saltzman A, Birol E, Bouis HE, Boy E, De MFF, Islam Y, Pfeiffer WH (2013). Biofortification: progress toward a more nourishing future. Glob Food Secur.

[15] Jones G, Steketee RW, Black RE, Bhutta ZA, Morris SS (2003). Bellagio child survival study group. How many child deaths can we prevent this year?. Lancet.

[16] Chattha MU, Hassan MU, Khan I, Chattha MB, Mahmood A, Chattha MU, Nawaz M, Subhani MN, Kharal M, Khan S (2017). Biofortification of wheat cultivars to combat zinc deficiency. Front Plant Sci.

[17] Hotz C (2009). The potential to improve zinc status through biofortification of staple food crops with zinc. IZiNCG Technical Document no 2. Food Nutr Bull.

[18] Transition in food consumption patterns. http://nutritionfoundationofindia.res.in/pdfpublication/Nutrition%20Transition%20in%20india1947-2007/6.%20Consumption%20expenditure.pdf. Accessed 21 Apr 2017.

[19] Cakmak I (2008). Enrichment of cereal grains with zinc: agronomic or genetic biofortification?. Plant Soil.

[20] Zhao FJ, McGrath SP (2009). Biofortification and phytoremediation. Curr Opin Plant Biol.

[21] Bouis HE, Hotz C, McClafferty B, Meenakshi JV, Pfeiffer WH (2011). Biofortification: a new tool to reduce micronutrient malnutrition. Food Nutr Bull.

[22] Sazawal S, Dhingra U, Dhingra P, Hiremath G, Sarkar A, Dutta A, Menon VP, Black RE (2010). Micronutrient fortified milk improves iron status, anemia and growth among children 1-4 years: a double masked, randomized, controlled trial. PLoS One.

[23] Gibson RS, Ferguson EL. An interactive 24-hour recall for assessing the adequacy of iron and zinc intakes in developing countries. Harvest Plus Technical Monograph 8. Washington (DC) and Cali (Colombia): International Food Policy Research Institute, and International Center for Tropical Agriculture; 2008. p. 1-160.

[24] Sazawal S, Black RE, Dhingra U, Dutta A, Deb S, Dhingra P (2015). Identifying iron deficiency anemia in developing country community settings: red cell distribution width with hemoglobin as an investigative tool in public health. J Pediatr Child Nutr.

[25] Aggarwal R, Sentz J, Miller MA (2007). Role of zinc administration in prevention of childhood diarrhea and respiratory illnesses: a meta-analysis. Pediatrics.

[26] Rosado JL, Hambidge KM, Miller LV, Garcia OP, Westcott J, Gonzalez K (2009). Quantity of zinc absorbed from wheat in adult women is enhanced by biofortification. J Nutr.

[27] Kodkany BS, Bellad RM, Mahantshetti NS, Westcott JE, Krebs NF, Kemp JF (2013). Biofortification of pearl millet with iron and zinc in a randomized controlled trial increases absorption of these minerals above physiologic requirements in young children. J Nutr.

[28] Chomba E, Westcott CM, Westcott JE, Mpabalwani EM, Krebs NF, Patinkin ZW (2015). Zinc absorption from biofortified maize meets the requirements of young rural Zambian children. J Nutr.

[29] Lu K, Li L, Zheng X, Zhang Z, Mou T, Hu Z (2008). Quantitative trait loci controlling Cu, Ca, Zn, Mn and Fe content in rice grains. J Genet.

[30] Islam MM, Woodhouse LR, Hossain MB, Ahmed T, Huda MN, Ahmed T (2013). Total zinc absorption from a diet containing either conventional rice or higher-zinc rice does not differ among Bangladeshi preschool children. J Nutr.

[31] Zou CQ, Zhang YQ, Rashid A, Ram H, Savasli E, Arisoy RZ (2012). Biofortification of wheat with zinc through zinc fertilization in seven countries. Plant Soil.

