# Supplementary material for: Efficacy of high zinc biofortified wheat in improvement of micronutrient status, and prevention of morbidity among preschool children and women - a double masked, randomized, controlled trial
Source: Nutr J. 2018 Sep 15;17:86. doi: 10.1186/s12937-018-0391-5 (PMC6139156; doi:10.1186/s12937-018-0391-5)
Supplement: Supplementary file 1 — Table S1. Zinc content of the study wheat flour. Table S2. Phytic acid content of the study wheat flour. (PDF 76 kb) [file 12937_2018_391_MOESM1_ESM.pdf]

Additional file Table S1 Zinc content of the study wheat flour

S. No.	Study Flour codes	Batch wise zinc content (mg/kg) of wheat flour																	
		1 (15 Jan 2014)	2 (19 May 2014)	3 (27 Jun 2014)	4 (2 Aug 2014)	5 (28 Aug 2014)	6 (25 Sep 2014)	7 (25 Oct 2014)	8 (10 Nov 2014)	9 (9 Dec 2014)	10 (20 Dec 2014)	11 (19 Jan 2015)	12 (13 Feb 2015)	13 (26 Feb 2015)	14 (17 Mar 2015)	15 (17 Apr 2015)	16 (25 May 2015)	17 (2 July 2015)	18 (3 Aug 2015)
1	A77582167	32.4	36.6	26	27.7	29	28.2	32	28	32.4	33.9	31.8	29.8	30	28.8	30.2	29.3	28.9	27.8
2	B77879005	34.8	36.1	31.8	34.3	32.1	36.3	32.2	32.9	32.9	29.5	32	32.4	25.2	32.6	29.8	33.9	34.1	31.8
3	C78827329	32.2	33.9	25.8	34.7	29.2	31.1	31.4	30.5	22.9	30.5	29.2	27.2	31.1	30.5	26.4	32.9	33.3	29.2
4	D74642767	26.4	23.8	29.5	33.5	31.2	31.6	21.3	29.8	26.8	32.1	30	32.8	30.7	39.3	28.7	31.6	31.6	30.3
5	E83530433	19.7	20.4	19.8	20.4	19.7	18.9	18.8	20.3	18.8	22.1	22.2	20.6	20.4	20.5	20.6	20.8	22.6	20.5
6	F81213325	19.6	21.7	20.7	22.9	19.5	18.8	18.6	19.3	19.8	18.2	25.1	21.4	20	19.4	19.8	21.1	22.2	20.9
7	G86155432	20	24.2	20.3	20.3	21.2	19.6	18.8	21.3	20	20.9	23.2	21.6	19.8	25.5	19.4	21.8	22.6	22.4
8	H87240917	20.5	24.1	27.1	21.2	21.4	19.7	19	18.7	21.5	24.9	22.9	22.7	20.6	19.1	21.4	21.9	22.3	20.5

Table S2 Phytic acid content of the study wheat flour

S. No.	Study Flour codes	Batch wise Phytic ACID (% by mass) of wheat flour																	
		1 (15 Jan 2014)	2 (19 May 2014)	3 (27 Jun 2014)	4 (2 Aug 2014)	5 (28 Aug 2014)	6 (25 Sep 2014)	7 (25 Oct 2014)	8 (10 Nov 2014)	9 (9 Dec 2014)	10 (20 Dec 2014)	11 (19 Jan 2015)	12 (13 Feb 2015)	13 (26 Feb 2015)	14 (17 Mar 2015)	15 (17 Apr 2015)	16 (25 May 2015)	17 (2 July 2015)	18 (3 Aug 2015)
1	81213325	0.3	0.4	0.3	0.3	0.4	0.2	0.3	0.5	0.3	0.3	0.3	0.3	0.4	0.3	0.4	0.2	0.2	0.6
2	86155432	0.4	0.4	0.3	0.2	0.4	0.3	0.3	0.4	0.4	0.4	0.3	0.3	0.3	0.4	0.4	0.3	0.3	0.4
3	83530433	0.3	0.3	0.3	0.2	0.4	0.2	0.3	0.3	0.4	0.3	0.3	0.2	0.4	0.3	0.5	0.3	0.2	0.5
4	87240917	0.4	0.3	0.5	0.3	0.3	0.2	0.3	0.5	0.4	0.4	0.3	0.3	0.4	0.3	0.5	0.2	0.2	0.4
5	74642767	0.4	0.4	0.4	0.3	0.4	0.2	0.3	0.4	0.4	0.3	0.3	0.4	0.4	0.5	0.4	0.2	0.4	0.5
6	78827329	0.5	0.4	0.6	0.3	0.3	0.3	0.5	0.5	0.4	0.4	0.3	0.4	0.4	0.3	0.5	0.3	0.4	0.5
7	77879005	0.6	0.4	0.3	0.3	0.4	0.3	0.4	0.4	0.4	0.4	0.3	0.3	0.4	0.4	0.4	0.4	0.3	0.5
8	77582167	0.5	0.6	0.3	0.4	0.4	0.2	0.4	0.5	0.4	0.3	0.3	0.3	0.4	0.4	0.5	0.3	0.3	0.6
